# Excessive spinal glutamate transmission is involved in oxaliplatin-induced mechanical allodynia: a possibility for riluzole as a prophylactic drug

**DOI:** 10.1038/s41598-017-08891-1

**Published:** 2017-08-29

**Authors:** Shota Yamamoto, Soichiro Ushio, Nobuaki Egashira, Takehiro Kawashiri, Shohei Mitsuyasu, Hitomi Higuchi, Nana Ozawa, Ken Masuguchi, Yuko Ono, Satohiro Masuda

**Affiliations:** 1 Department of Clinical Pharmacology and Biopharmaceutics, Graduate School of Pharmaceutical Sciences, Kyushu University, 3-1-1 Maidashi, Higashi-ku, Fukuoka, 812-8582 Japan; 2 Department of Pharmacy, Kyushu University Hospital, 3-1-1 Maidashi, Higashi-ku, Fukuoka, 812-8582 Japan

## Abstract

Oxaliplatin, a chemotherapy medication, causes severe peripheral neuropathy. Although oxaliplatin-induced peripheral neuropathy is a dose-limiting toxicity, a therapeutic strategy against its effects has not been established. We previously reported the involvement of *N*-methyl-D-aspartate receptors and their intracellular signalling pathway in oxaliplatin-induced mechanical allodynia in rats. The aim of this study was to clarify the involvement of spinal glutamate transmission in oxaliplatin-induced mechanical allodynia. *In vivo* spinal microdialysis revealed that the baseline glutamate concentration was elevated in oxaliplatin-treated rats, and that mechanical stimulation of the hind paw markedly increased extracellular glutamate concentration in the same rats. In these rats, the expression of glutamate transporter 1 (GLT-1), which plays a major role in glutamate uptake, was decreased in the spinal cord. Moreover, we explored the potential of pharmacological therapy targeting maintenance of extracellular glutamate homeostasis. The administration of riluzole, an approved drug for amyotrophic lateral sclerosis, suppressed the increase of glutamate concentration, the decrease of GLT-1 expression and the development of mechanical allodynia. These results suggest that oxaliplatin disrupts the extracellular glutamate homeostasis in the spinal cord, which may result in neuropathic symptoms, and support the use of riluzole for prophylaxis of oxaliplatin-induced mechanical allodynia.

## Introduction

Oxaliplatin has widely been used for the treatment of solid cancers such as colorectal cancer and gastric cancer. However, it causes severe peripheral neuropathy. This neuropathy, which is characterized by sensory and motor dysfunction, is a dose-limiting toxicity and a major clinical problem in oxaliplatin chemotherapy^1, 2^. However, the mechanisms underlying oxaliplatin-induced peripheral neuropathy remain unclear, and therapeutic strategies to prevent oxaliplatin-induced neuropathy have not been established^3, 4^.

Excessive activation of glutamate receptors, in particular *N*-methyl-D-aspartate receptors (NMDAR), in the spinal cord is a hallmark mechanism of neuropathic pain^5–7^. Accumulating evidence has indicated that activation of NMDAR is governed by three essential factors: the amount of synaptically released glutamate, the rate at which glutamate is removed by glutamate transporters (GTs), and the properties of postsynaptic NMDAR^7–9^.

The homeostasis of extracellular glutamate at the synaptic cleft is ensured by GTs located in the plasma membranes of both glial cells and neurons^10^. Three types of GTs exist in the spinal cord: glutamate/aspartate transporter [glial glutamate transporter (GLAST)/excitatory amino acid transporter 1 (EAAT1)], glutamate transporter 1 (GLT-1)/EAAT2 and excitatory amino acid carrier 1 (EAAC1)/EAAT3^9^. It has been reported that pain hypersensitivity induced by peripheral nerve injury and chemotherapy (e.g., paclitaxel and bortezomib) is associated with downregulation of GTs in the spinal dorsal horn^11–14^. In addition, some studies have shown that therapeutic agents, such as riluzole and ceftriaxone, which are known as positive GTs activity regulators^15, 16^, attenuate inflammatory and neuropathic pain in rodents^12, 17^.

We have previously reported that repeated administration of oxaliplatin induced cold allodynia in the early phase and mechanical allodynia in the late phase in rats^18^. Oxaliplatin is metabolized to oxalate and dichloro(1,2-diaminocyclohexane)platinum^19^. We have reported that oxalate and platinum metabolites were involved in the cold allodynia and mechanical allodynia, respectively^18^. We have also reported that Ca^2+^ channels/nuclear factor of activated T-cell/transient receptor potential melastatin 8 pathway is involved in the oxaliplatin-induced cold allodynia^20^. On the other hand, we have reported that the intrathecal administration of NMDAR antagonists and selective NMDAR subtype 2B antagonists reverse oxaliplatin-induced mechanical allodynia^21^. Moreover, we have shown that the NMDAR-Ca^2+^/calmodulin-dependent protein kinase II pathway is involved in oxaliplatin-induced mechanical allodynia^22^. Thus, oxaliplatin-induced mechanical allodynia seems be related to glutamate neuronal transmission. Recently, it has been reported that P2 × 7 receptor-dependent glutamate release is enhanced in synaptosomes purified from cerebral cortex of oxaliplatin-treated rats^23^. However, whether oxaliplatin induces an abnormality of extracellular glutamate concentration in the spinal cord remains unclear.

In the present study, we attempted to elucidate above questions using *in vivo* spinal microdialysis, and revealed that the glutamate concentration in the cerebrospinal fluid of the lumbar spinal cord was increased in oxaliplatin-treated rats. Some agents (riluzole and ceftriaxone) that modulate glutamate concentration within the central nervous system are gaining attention in the field of neurodegenerative disease for their neuroprotective effects^24, 25^. Therefore, we used riluzole to explore the therapeutic potential for targeting the maintenance of spinal glutamate homeostasis to prevent oxaliplatin-induced mechanical allodynia. Our results provide support for the potency of riluzole as a prophylaxis for oxaliplatin-induced mechanical allodynia.

## Results

### Increase of glutamate, but not γ-aminobutyric acid (GABA), concentration in the cerebrospinal fluid

**Figure 1:**
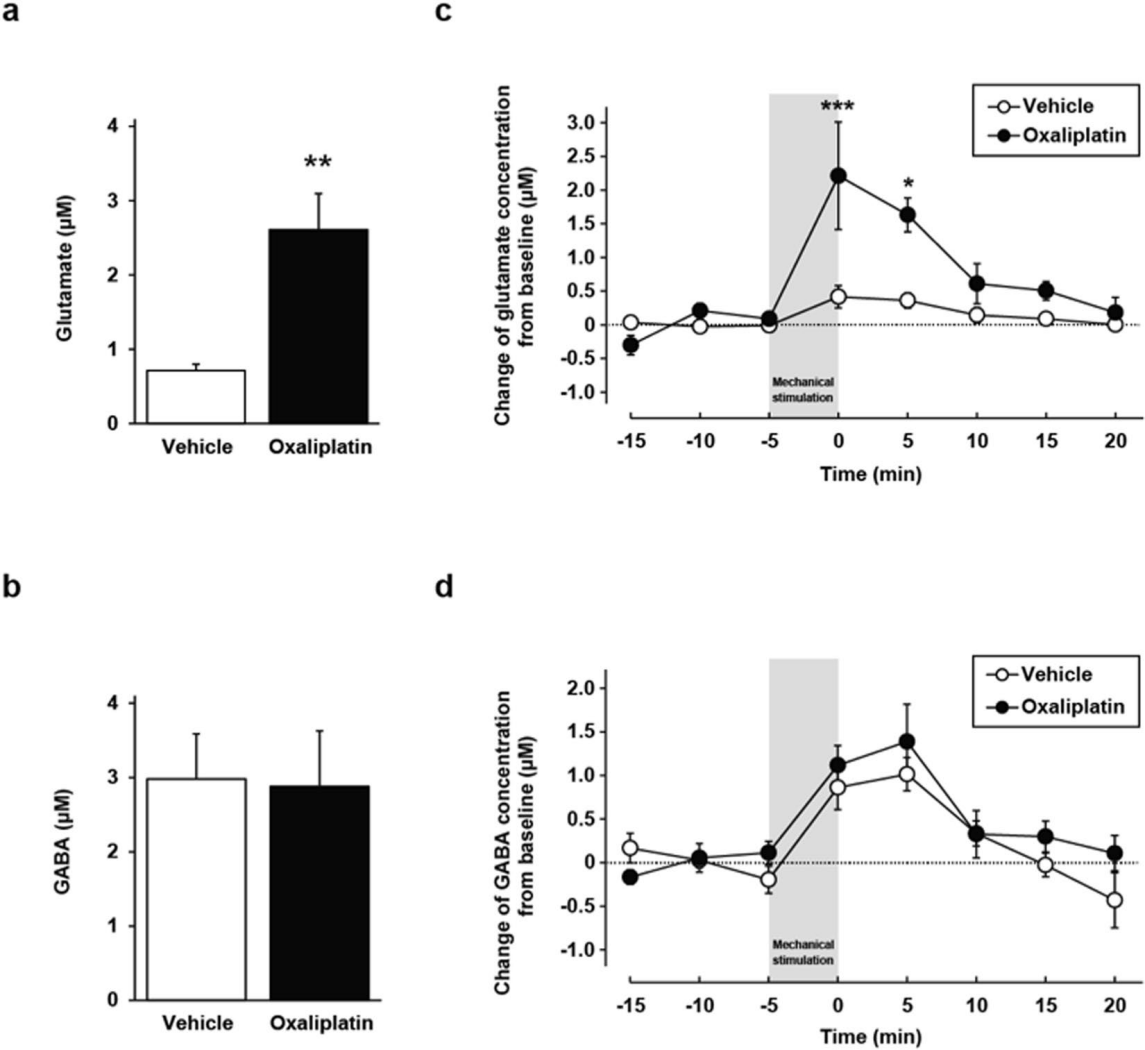
Glutamate and γ-aminobutyric acid (GABA) concentrations in the cerebrospinal fluid of the lumbar spinal cord (L4–L6). (**a**,**b**) Baseline concentrations of glutamate (**a**) and GABA (**b**). The mean concentration for the first three dialysate samples from the beginning of sample collection was defined as the baseline concentration. Values are expressed as the mean ± SEM (n = 4–6, **P < 0.01 vs. vehicle). (**c**,**d**) The mechanical stimulation-induced increase of extracellular glutamate (**c**) and GABA (**d**) from baseline. Values are expressed as the mean ± SEM (n = 4–6, *P < 0.05, ***P < 0.001 vs. vehicle).

Using low-invasive *in vivo* spinal microdialysis, we evaluated the glutamate concentration in the cerebrospinal fluid of the lumbar spinal cord (L4–L6). The basal glutamate concentration in oxaliplatin-treated rats was significantly higher than that in vehicle-treated rats on day 26 (vehicle: 0.71 ± 0.08 µM, oxaliplatin: 2.61 ± 0.48 µM, Fig. 1a). In contrast, there was no difference in the basal GABA concentration between the two groups (vehicle: 2.98 ± 0.60 µM, oxaliplatin: 2.88 ± 0.75 µM, Fig. 1b).

In the condition of mechanical allodynia, there is a possibility that presynaptic glutamate release is enhanced. Hence, we tested the change of glutamate concentration from baseline when hind paws were exposed to a mechanical stimulus for 5 min. In vehicle-treated rats, the mechanical stimulus significantly increased the extracellular glutamate concentration from baseline (0.04 ± 0.03 µM at −15 min, −0.03 ± 0.03 µM at −10 min, −0.01 ± 0.02 µM at −5 min and 0.42 ± 0.17 µM at 0 min; −15 min vs. 0 min; *P* < 0.01, −10 min vs. 0 min; *P* < 0.01, −5 min vs. 0 min; *P* < 0.05, Fig. 1c), which was enhanced in oxaliplatin-treated rats (2.21 ± 0.80 µM at 0 min, Fig. 1c). However, there was no significant difference in the change of GABA concentration from baseline between the two groups (vehicle: 1.01 ± 0.19 µM, oxaliplatin: 1.39 ± 0.43 µM, Fig. 1d). Next, we evaluated the change of glutamate concentration when 5% (v/v) formalin was injected subcutaneously into the plantar skin surface of the hind paw, to observe how the degree of glutamate increase induced by a mechanical stimulus compared to changes induced by a noxious formalin stimulus. After formalin injection, the glutamate concentration was equally elevated in vehicle- and oxaliplatin-treated rats (vehicle: 1.13 ± 0.78 µM, oxaliplatin: 1.54 ± 0.72 µM, Supplementary Fig. 2). Furthermore, the degree of elevation was similar to that induced by the mechanical stimulus in oxaliplatin-treated rats. These results suggest that oxaliplatin treatment changes the reactivity to mechanical stimulation to enhance the increase in glutamate concentration, which is capable of inducing mechanical allodynia.

### Downregulation of glutamate transporters in the spinal dorsal horn

**Figure 2:**
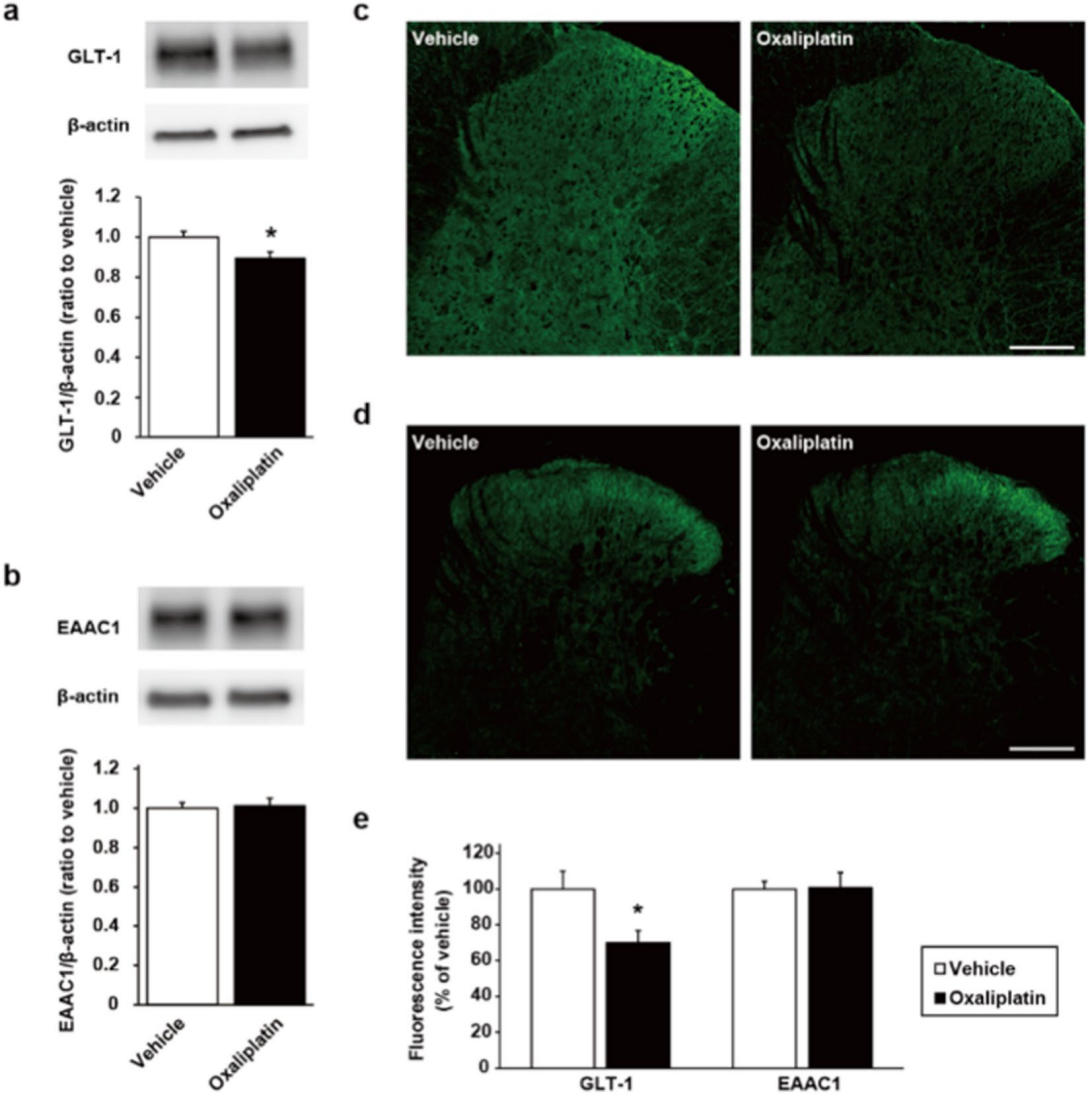
Glutamate transporters expression in the spinal dorsal horn. (**a**,**c**) Analysis of GLT-1 expression by western blot (**a**: n = 8) and by immunohistochemistry (**c**) on day 28 (scale bar, 200 µm). (**b**,**d**) Analysis of EAAC1 expression by western blot ((**b**) n = 6) and by immunohistochemistry (**d**) on day 28 (scale bar, 200 µm). (**e**) Quantification of fluorescence intensity of immunohistochemistry in the spinal lamina I–II. Values are expressed as the mean ± SEM (GLT-1: n = 6, EAAC1: n = 8, *P < 0.05 vs. vehicle). Full-length blots are shown in Supplementary Fig. 4.

To explore potential mechanisms involved in the oxaliplatin-induced increase of glutamate concentration, we evaluated the expression levels of GTs in the spinal dorsal horn by western blot analysis. On day 7, oxaliplatin treatment did not alter the protein levels of GTs (Supplementary Fig. 3). However, the protein level of GLT-1 in the spinal dorsal horn was significantly reduced in oxaliplatin-treated rats compared with vehicle-treated rats on day 28, whereas there were no differences between the two groups in expression levels of EAAC1 (Fig. 2a,b). We also examined the immunoreactivities of GLT-1 and EAAC1 in the spinal dorsal horn. Consistent with the western blot analysis, the immunoreactivity in the superficial spinal dorsal horn of GLT-1, but not EAAC1, was reduced in oxaliplatin-treated rats 4 weeks after the first injection of oxaliplatin (Fig. 2c,d,e). These findings suggest that the downregulation of GLT-1 expression induces the increase of glutamate concentration in the synaptic cleft, which results in oxaliplatin-induced mechanical allodynia.

### Riluzole suppresses the development of oxaliplatin-induced mechanical allodynia

**Figure 3:**
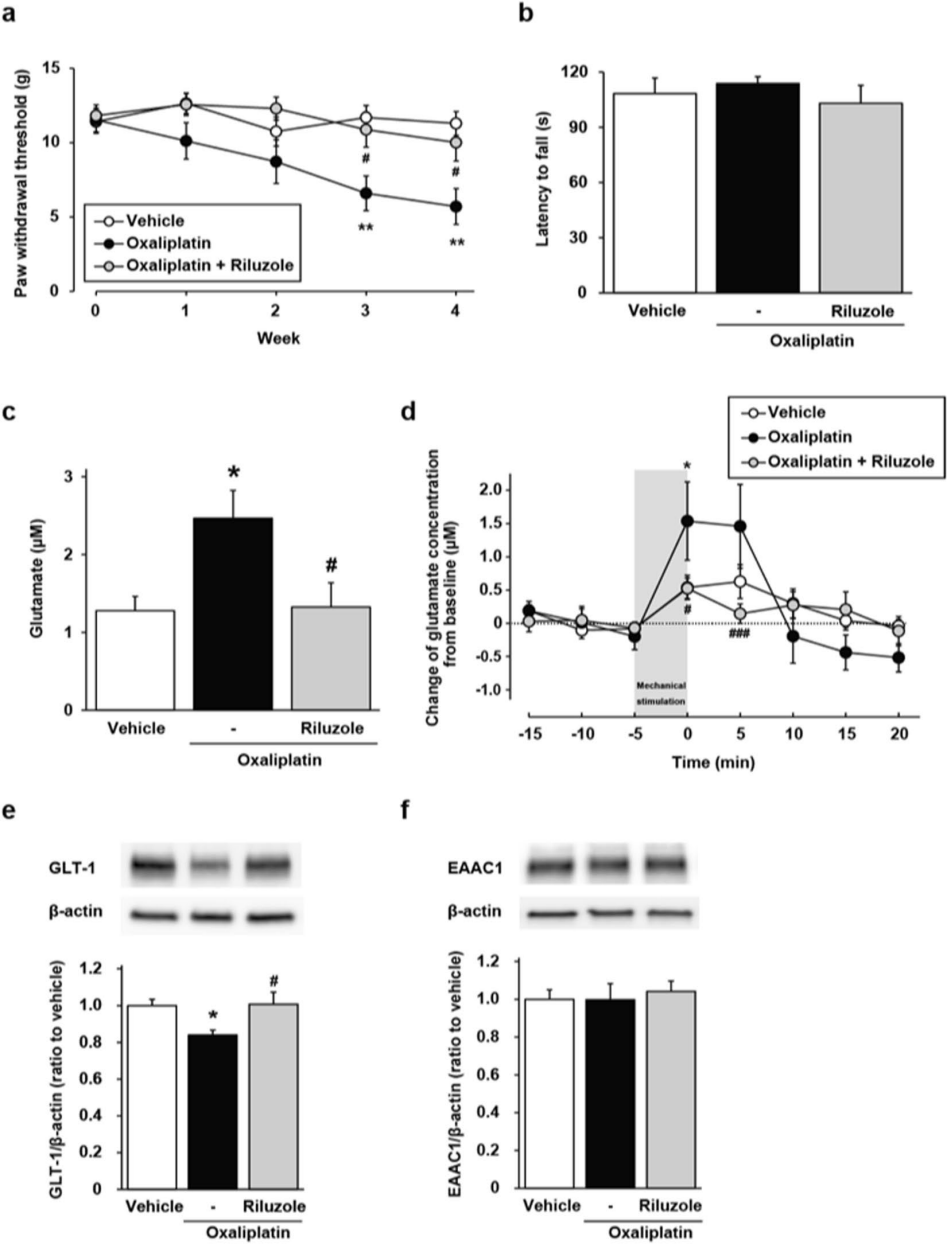
Effect of riluzole on oxaliplatin-induced alterations. Oxaliplatin (4 mg/kg, i.p.) was administered twice a week for 4 weeks (days 1, 2, 8, 9, 15, 16, 22 and 23). Riluzole (12 mg/kg, p.o.) was administered once a day for 27 days. (**a**,**b**) Effect of riluzole on oxaliplatin-induced mechanical allodynia (**a**: von Frey test) and motor coordination (**b**: rota-rod test). Values are expressed as the mean ± SEM (**a**) n = 10–11, **P < 0.01 vs. vehicle, ^#^ *P* < 0.05 vs. oxaliplatin, (**b**) n = 8). (**c**,**d**) Effect of riluzole on oxaliplatin-induced increase of basal glutamate concentration (**c**), and that in response to mechanical stimulation (**d**) in the cerebrospinal fluid of the lumbar spinal cord (L4–L6). Values are expressed as the mean ± SEM (n = 5–8, *P < 0.05 vs. vehicle, ^#^ *P* < 0.05, ^###^ *P* < 0.001 vs. oxaliplatin). (**e**,**f**) Effect of riluzole on protein expression of GLT-1 (**e**), and EAAC1 (**f**) in the spinal dorsal horn on day 28. Values are expressed as the mean ± SEM (n = 8–11, *P < 0.05 vs. vehicle, ^#^ *P* < 0.05 vs. oxaliplatin). Full-length blots are shown in Supplementary Fig. 4.

We next investigated whether the pharmacological approach with riluzole, a GTs activator, has a protective effect on oxaliplatin-induced hypersensitivity to mechanical stimulation. Oxaliplatin [4 mg/kg, intraperitoneally (i.p.)] significantly decreased the paw withdrawal threshold in a cumulative dose-dependent manner compared with the vehicle-treated rats (Fig. 3a). Co-administration of riluzole [12 mg/kg, per os (p.o.)] prevented the decrease in the paw withdrawal threshold in oxaliplatin-treated rats. Moreover, we examined the influence of repeated administration of riluzole on motor coordination in a rota-rod test; there were no differences between the groups (Fig. 3b).

### Riluzole prevents oxaliplatin-induced disruption of the extracellular glutamate homeostasis in the spinal cord

*In vivo* spinal microdialysis showed that repeated administration of riluzole notably suppressed the oxaliplatin-induced increase of basal glutamate concentration and enhancement of glutamate release induced by mechanical stimulation (Fig. 3c,d). Furthermore, we examined the effects of riluzole on the downregulation of GLT-1 induced by oxaliplatin. In riluzole co-treated rats, the protein level of GLT-1 remained at a comparable level to vehicle-treated rats (Fig. 3e), and riluzole co-treatment did not affect the expression of EAAC1 (Fig. 3f). These results indicate that pharmacological therapy with riluzole may help maintain glutamate homeostasis in the synaptic cleft of the spinal dorsal horn during treatment with oxaliplatin.

### Riluzole does not affect anti-tumor activity of oxaliplatin *in vitro* and *in vivo* assay

**Figure 4:**
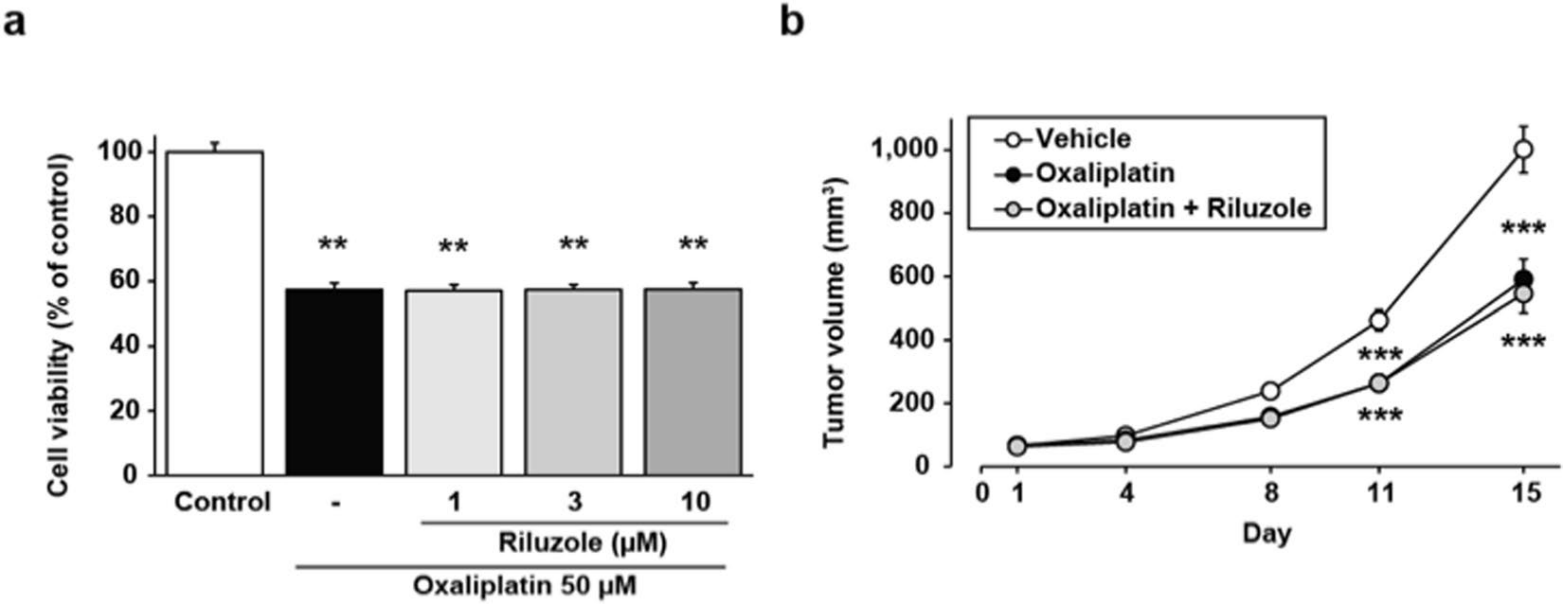
Effect of riluzole on anti-tumour activity of oxaliplatin in *in vitro* and *in vivo* assays. (**a**) C-26 cells were exposed to oxaliplatin (50 µM) for 24 h in the presence or absence of various concentrations (1, 3 or 10 µM) of riluzole. (**b**) C-26 cells-implanted mice were treated with oxaliplatin (6 mg/kg, i.p.) twice a week (days 1, 2, 8 and 9) and riluzole (18 mg/kg, p.o.) once a day for 2 weeks. The tumour volumes were calculated as follows: Volume (mm^3^) = π/6 × Thickness (mm) × Length (mm) × Width (mm). Values are expressed as the mean ± SEM ((**a**) n = 4, ***P < 0.001 vs. control, (**b**) n = 10–11, ***P < 0.001 vs. vehicle).

Finally, to confirm whether riluzole could be used as a protective medication against oxaliplatin-induced neuropathic pain, we evaluated the effects of riluzole on anti-tumour activity of oxaliplatin with both *in vitro* and *in vivo* experiments. In cultured C-26 cells, the exposure to oxaliplatin (50 µM) significantly inhibited cell growth, and riluzole (1–10 µM) had no effect on the oxaliplatin-induced cytotoxicity in the cell line (Fig. 4a). In agreement with the *in vitro* experiment, oxaliplatin (6 mg/kg, i.p.) in tumour cells-implanted mice prominently inhibited the increase of tumour volumes compared with vehicle, and riluzole (18 mg/kg, p.o.) had no effect on the oxaliplatin-induced inhibition of tumour growth (Fig. 4b).

## Discussion

*In vivo* microdialysis is an essential method in neuroscience research for studying neurochemical events at the individual level without restraint and anaesthesia. Multiple studies have observed glutamate release in the spinal cord after noxious stimuli such as formalin or capsaicin^26, 27^. To the best of our knowledge, this is the first report to directly observe the mechanical stimulus-induced increase of extracellular glutamate levels near the lumbar spinal cord in unanaesthetised animals. Some previous reports have examined the basal level of neurotransmitter by means of *in vivo* spinal microdialysis in neuropathic animals. Yoshizumi and colleagues have reported that spinal nerve ligation induces an increase of basal glutamate and decrease of basal GABA concentrations in the spinal dorsal horn under anaesthesia^28, 29^. In contrast, it has been reported that basal glutamate concentration is unchanged in a chronic constriction injury model^26^, and that basal glutamate concentration is decreased and basal GABA concentration is increased in a streptozotocin-induced diabetic rat model^30^. Thus, there is no consensus regarding basal levels of neurotransmitter in the spinal cord in neuropathic conditions. In this study, we found that oxaliplatin treatment induced an increase of basal glutamate concentration, but did not alter the GABA concentration in the spinal cord compared with vehicle treatment. Cumulative evidence suggests that increased extracellular glutamate and activation of glutamate receptors promotes synaptic plasticity, and that spinal neuronal plasticity plays a critical role in the persistence of pain^6, 31^. Taken together, our previous studies and present results suggest that excessive glutamate in the spinal cord activates NMDAR, which then leads to the development of oxaliplatin-related mechanical allodynia^21, 22^.

Interestingly, our results from the present study showed that mechanical stimulus-induced glutamate release was significantly enhanced in oxaliplatin-treated rats compared with vehicle-treated rats, which corresponds to the development of mechanical hypersensitivity. In contrast, formalin stimulation induced glutamate release equally in the two groups. Primary sensory afferent axons can be distinguished by their response properties^32, 33^. Our data indicate that oxaliplatin treatment enhances the activity of mechanoreceptive afferents, but not the activity of afferents that respond to chemical irritation.

Although GABA neuronal system basically has an inhibitory role in control of pain, it has been reported that GABA neurons are shifted to excitatory function in neuropathic pain animal model^34^. In the present study, we did not observe changes in basal GABA levels in the spinal cord of oxaliplatin-treated rats. Moreover, the mechanical stimulus-induced GABA release in oxaliplatin-treated rats was similar to that of vehicle-treated rats. However, we have not measured the GABA concentration in the synaptic cleft, so the possibility of involvement of GABA neuronal system in oxaliplatin-induced mechanical allodynia cannot be ruled out.

Glutamate homeostasis is maintained by a balance between release and uptake. It has been reported that nerve injury induces downregulation of GTs in the spinal cord, which leads to pain hypersensitivity via excessive activation of NMDAR^12, 35^. GLT-1 is a major glutamate transporter in the central nervous system^10^. Among some GTs, the expression level of GLT-1 is correlated highly with development of pain behaviours in some neuropathic animal models^9^. However, conflicting findings have been reported for EAAC1. It has been reported that the expression of EAAC1 in the spinal cord is decreased after chronic constriction injury^12, 36^. In contrast, Cirillo *et al*. have demonstrated an upregulation of EAAC1 in spared nerve injury^37, 38^. In our present study, we observed that GLT-1 expression was decreased, but EAAC1 expression was unchanged in the spinal dorsal horn 4 weeks after the first injection of oxaliplatin. Our results indicate that the downregulation of GLT-1 in the spinal dorsal horn may play a role in the accumulation of extracellular glutamate, which contributes to the high level of basal glutamate concentration in oxaliplatin-treated rats. However, the degree of downregulation of GLT-1 was smaller than the degree of increase in extracellular glutamate concentration in this study. Hu *et al*. have reported that glutamate uptake activity was markedly decreased as compared to the decrease in the spinal GLT-1 expression in a rat sciatic nerve chronic constrictive nerve injury model^39^. From these facts, the degree of increase in extracellular glutamate concentration may not necessarily coincide with the degree of decrease in the expression of GLT-1. One of the reasons may be the involvement of other GTs such as GLAST. Therefore, further investigations are needed to fully understand the mechanisms of the marked increase in extracellular glutamate concentration by oxaliplatin.

In this study, we showed that co-treatment with riluzole, which positively regulates GT activity^15^, inhibited the downregulation of GLT-1 and the increase of glutamate concentration in the spinal cord. Riluzole also suppressed the decreased paw withdrawal threshold of oxaliplatin-treated rats in the von Frey test. Furthermore, repeated administration of riluzole (12 mg/kg, once a day for 4 weeks) had no effect on motor coordination. Hence, riluzole prevented oxaliplatin-induced mechanical allodynia distinct from an impairment of motor coordination. We carried out all *in vivo* experiments with riluzole about 24 hours after administration of procedures the previous day, such as behavioural tests, collections of dialysates via microdialysis probe and collections of spinal cord tissues. A pharmacokinetics study has shown that when rats repeatedly receive riluzole, the steady-state concentration of the drug is about one-eighth of the maximum concentration in the spinal cord tissues^40^. Therefore, it is plausible that repeated administration of riluzole suppresses the development of oxaliplatin-induced mechanical allodynia via maintenance of GT expression and activity, rather than through the effects of its steady-state levels. Although it has been reported that riluzole treatment has no efficacy for patients who have acute inflammatory pain or chronic neuropathic pain, these studies tested riluzole as a palliative agent, not as a prophylactic^41, 42^. Our results suggest that riluzole exerts its effectiveness for the treatment of neuropathic pain when it is used as a prophylactic agent.

In the present study, we found that riluzole prevented the downregulation of GLT-1 by oxaliplatin treatment. We have previously reported that repeated administration of oxaliplatin induces apoptosis of dorsal root ganglia neuron and axonal degeneration in rat sciatic nerve^43^. It has been reported that nerve injury leads to downregulation of GLT-1, and that co-culturing of astrocytes with injured neurons downregulates GLT-1^44, 45^. In addition, the exposure of riluzole has been reported to facilitate cell survival and neurite outgrowth in primary culture of dorsal root ganglia neurons^46^. A possible mechanism from these facts was that oxaliplatin caused downregulation of GLT-1 via nerve injury and riluzole inhibited the downregulation of GLT-1 by protecting neuronal damage. Further investigation is necessary for elucidation of this mechanism.

In this study, formalin treatment as nociceptive stimulus caused a marked increase in extracellular glutamate levels in vehicle and oxaliplatin-treated rats. In mechanical stimulus, a marked increase in extracellular glutamate concentration was observed in oxaliplatin-treated rats, and the degree of increase by mechanical stimulus was similar to that by formalin treatment. Moreover, the marked increase in extracellular glutamate concentration by mechanical stimulus was inhibited by riluzole suppressing oxaliplatin-induced mechanical allodynia. Therefore, the mechanical stimulus may be painful for oxaliplatin-treated rats.

In conclusion, we directly demonstrated, for the first time, that oxaliplatin treatment increased both basal and mechanical stimulation-induced levels of extracellular glutamate concentrations in the spinal cord. We also found that downregulation of GLT-1 is one likely cause of excessive glutamate in the spinal cord. Furthermore, repeated administration of riluzole suppressed mechanical allodynia and downregulation of GLT-1 induced by oxaliplatin, which resulted in normal glutamate transmission in the spinal cord. These results indicate that spinal glutamate transmission plays an important role in the development of oxaliplatin-induced mechanical allodynia, and that pharmacological therapy that targets maintenance of activity or expression of GLT-1, such as treatment with riluzole, may be useful as prophylaxis for oxaliplatin-induced neuropathic pain.

## Methods

### Animals

Male Sprague-Dawley rats (Kyudo Co., Saga, Japan) aged 6 weeks were used for the oxaliplatin-induced mechanical allodynia. Male BALB/c mice (Kyudo Co.) aged 7–8 weeks were used for the *in vivo* tumour growth model. They were housed individually or in groups of four per cage, with lights on from 7:00 to 19:00 h. Animals had free access to food and water in their home cages. All experiments were approved by the Experimental Animal Care and Use Committee of Kyushu University according to the National Institutes of Health guidelines, and we followed the International Association for the Study of Pain Committee for Research and Ethical Issues guidelines for animal research^47^.

### Drugs

Oxaliplatin (Elplat^®^) was obtained from Yakult Co., Ltd. (Tokyo, Japan), and was dissolved in 5% (w/v) glucose solution. To establish the oxaliplatin-induced mechanical allodynia, oxaliplatin (4 mg/kg, i.p.) or vehicle (5% (w/v) glucose solution) was administered twice a week for 4 weeks (days 1, 2, 8, 9, 15, 16, 22 and 23). Riluzole was purchased from Tokyo Chemical Industry Co., Ltd. (Tokyo, Japan) and was dissolved in 0.1 M hydrochloric acid (HCl) according to previous reports^48^. Riluzole (12 mg/kg, p.o.) or 1 mM HCl as vehicle solution was administered once a day for 27 days from day 1. The injection volume was 1 mL/kg. The doses of oxaliplatin and riluzole were chosen based on previous reports^18, 49, 50^.

### Behavioral analysis

Mechanical allodynia was assessed by the von Frey test. Each rat was placed in a wire mesh cage and habituated for 30–60 min before testing. Calibrated von Frey filaments (0.4–15 g, The Touch Test Sensory Evaluator Set; Linton Instrumentation, Norfolk, UK) were applied to the mid-plantar skin of each hind paw. The 50% paw withdrawal threshold was determined using the up-down method^51^. Rota-rod test was performed to assess motor coordination. Rats were placed on a rotating rod (Muromachi Kikai Co., Ltd., Tokyo, Japan) and the latency to fall was measured for up to 2 min according to the method described previously^52^.

### *In vivo* spinal microdialysis

The method for low-invasive *in vivo* spinal microdialysis and probe construction was modified from previous studies^53, 54^. The methods have been divided into three types based on the location of the microdialysis probe: transversal, concentric, and intrathecal^55^. In this study, we used the intrathecal approach as it is minimally-invasive. The dialysis probe was constructed of a 10.8 cm PE-10 tube and 1 cm cuprophan hollow fibre (inner diameter: 0.2 mm, outer diameter: 0.22 mm; Eicom, Kyoto, Japan) as an active zone. On day 23, rats were anesthetized with 2% (v/v) isoflurane and implanted with a microdialysis loop probe near the lumbar enlargement (L4–6) of the spinal cord via the atlanto-occipital region (Supplementary Fig. 1). After 34 days from the implantation, each rat was placed in a cage stuffed with wooden chips, and then the probe was perfused with Ringer’s solution (Na^+^: 147 mM, K^+^: 4 mM, Ca^2+^: 2.3 mM and Cl^−^: 155.6 mM) with a flow rate of 5 µL/min using the syringe pump (EP-60, Eicom). Collection tubes were connected to a dialysis probe by Microdialysis Teflon^®^ Tubing (JT-10-50). After 1 h of stabilization, the dialysates were collected 8 times every 5 min, and immediately frozen at −80 °C. The mean concentration during 15 min from the initiation of the experiment was defined as the baseline concentration.

After the end of the experiment, the probe was perfused with 2.5% (w/v) bromophenol blue for 10 min to confirm the location of the dialysis probe. The rats were deeply anaesthetised with 2% (v/v) isoflurane, and the lumbar spinal cord was removed. The placement of the dialysis membrane was confirmed by visual observation.

### Mechanical stimulation

After 15 min of the start of dialysates collection, each rat was placed in an acrylic box with a polypropylene-brush floor for 5 min and then returned to the cage.

### Measurements of glutamate and GABA

Cerebrospinal fluid dialysates were applied to a reversed-phase high-performance liquid chromatography electrochemical detection (HPLC-ECD). The HPLC system used in this study was a HTEC-MM with a C-18 ODS column; the EICOMPAK SC-50DS (2.1 × 150 mm; Eicom). The mobile phase was composed of 0.1 M phosphate buffer (pH 6.0), methanol (30% (v/v)), and EDTA 2Na (5 mg/L). Dialysate was derivatized with 2-mercaptoethanol and o-phthalaldehyde in 0.1 M carbonate buffer, and then injected into HPLC-ECD in volumes of 20 µL. A standard curve was made using known concentrations of glutamate and GABA. External standards were run to ensure accuracy and for calculations of concentration.

### Western blotting analysis

Rats were deeply anaesthetised and perfused with phosphate buffered saline (PBS). The dorsal horn of the L4–L5 spinal cord was quickly removed and homogenised in homogenisation buffer (20 mM Tris-HCl pH 7.4, 2 mM EDTA, 0.5 mM EGTA, 0.32 M sucrose, protease, and phosphatase inhibitors cocktails). Protein concentrations were determined by BCA Protein assay (Thermo Fisher Scientific, MA, USA). Protein lysates were diluted with 2 × Laemmli sample buffer containing 5% 2-mercaptoethanol and incubated at 95 °C. Proteins were separated by sodium dodecyl sulfate PAGE gel, and transferred electrophoretically to PVDF membranes. After blocking with blocking one (Nacalai Tesque, Kyoto, Japan), the membranes were incubated with primary antibodies: rabbit GLT-1 antibody (1:1,000, Cell Signaling Technology, MA, USA); rabbit EAAC1 antibody (1:1,000, Santa Cruz Biotechnology, CA, USA); anti-β-actin mouse monoclonal antibody (1:2,000, Sigma, MO, USA), and then incubated with HRP-conjugated secondary antibody (1:1,000, GE Healthcare UK Ltd., Buckinghamshire, UK). Signals were detected using ECL Western Blotting Detection Reagents (GE Healthcare UK Ltd.). The intensity of the bands was analysed using ImageJ.

### Immunohistochemistry

Rats were deeply anaesthetised with pentobarbital (100 mg/kg, i.p.) and perfused transcardially with PBS, followed by ice-cold 4% (w/v) paraformaldehyde. The L4–L6 spinal cord was removed and immersed in the fixative, and placed in 30% (w/v) sucrose solution at 4 °C. The transverse spinal cord sections were cut. The sections were incubated with primary antibody: anti-GLT-1 rabbit polyclonal antibody (1:500, Cell Signaling Technology) and anti-EAAC1 rabbit polyclonal antibody (1:500, Santa Cruz Biotechnology). After incubation, the sections were washed and incubated with anti-rabbit Alexa Fluor 488-conjugated secondary antibody (1:1000, Molecular Probes, OR, USA). Prepared slides were observed using a fluorescence microscope (BZ-9000; Keyence Corporation, Osaka, Japan).

### Cell culture

Murine colon adenocarcinoma 26 (C-26) cells were obtained from RIKEN (Saitama, Japan). The cells were grown in RPMI-1640 supplemented with penicillin-streptomycin and 10% fetal bovine serum. The cells were cultured at 37 °C in air supplemented with 5% CO_2_ under humidified conditions.

### Tumour cytotoxicity assay

The cell viability was assessed by WST-8 assay as described previously^52^. In brief, C-26 cells were seeded onto 24-well plates at a density of 1 × 10^4^ cells/well, 24 h before drug treatment. The cells were exposed to oxaliplatin (50 µM) and riluzole (1, 3 or 10 µM) for 24 h, and then the cells were incubated with WST-8 assay solution (Cell Counting Kit-8; Dojindo, Kumamoto, Japan) for 1 h. The incubation medium was transferred to 96-well flat-bottom plastic plates. The amount of WST-8 metabolite was measured from the absorbance at 450 nm using a Mithras LB940 multi-label microplate reader (Berthold Technologies, Bad Wildbad, Germany).

### Tumour growth analysis

C-26 cells (1.5 × 10^6^ cells per mouse in 30 µL PBS) were implanted subcutaneously in the hind paws of BALB/c mice. Three days after implantation of tumour cells, administration of drugs was started. Oxaliplatin (6 mg/kg) or vehicle (5% glucose solution) was administered i.p. twice a week for 2 weeks (days 1, 2, 8 and 9). Riluzole (18 mg/kg) or vehicle (HCl) was administered p.o. once a day for 14 days. The injection volume was 10 mL/kg. The doses of oxaliplatin and riluzole were chosen based on previous reports^56^. The tumour volumes were calculated as follows: Volume (mm^3^) = π/6 × Thickness (mm) × Length (mm) × Width (mm).

### Statistical analysis

Data were analysed using the Student’s *t*-test (Figs 1a,b and 2), two-way ANOVA with *post hoc* Bonferroni test (Figs 1c,d, 3a,d and 4b), or one-way ANOVA with *post hoc* Dunnett’s (Fig. 1c,d) or Tukey’s test (Figs 3b,c,e,f and 4a) using GraphPad Prism 4.03 software to determine differences among the groups. A probability level of *P* < 0.05 was accepted as statistically significant.

### Data Availability

The datasets generated during the current study are available from the corresponding author on reasonable request.

## Electronic supplementary material

Supplementary Figure
