# Supplementary material for: Excessive spinal glutamate transmission is involved in oxaliplatin-induced mechanical allodynia: a possibility for riluzole as a prophylactic drug
Source: Sci Rep. 2017 Aug 29;7:9661. doi: 10.1038/s41598-017-08891-1 (PMC5574967; doi:10.1038/s41598-017-08891-1)
Supplement: Supplementary file 1 — Supplementary Figure [file 41598_2017_8891_MOESM1_ESM.pdf]

Supplementary information

Excessive spinal glutamate transmission is involved in oxaliplatin-induced mechanical allodynia: a possibility of riluzole as a prophylactic drug

Shota Yamamoto, Soichiro Ushio, Nobuaki Egashira, Takehiro Kawashiri, Shohei Mitsuyasu, Hitomi Higuchi, Nana Ozawa, Ken Masuguchi, Yuko Ono, Satohiro Masuda

Supplementary Figure 1. Schematic illustration of the microdialysis loop probe-implanted rat.
Each rat was implanted with a microdialysis loop probe near by the lumbar enlargement (L4-6) of the spinal cord via the atlanto-occipital region.

Supplementary Figure 2. The formalin injection-induced change of extracellular glutamate and γ-aminobutyric acid (GABA) concentrations in the cerebrospinal fluid immediate lumbar spinal cord (L4-6).
5% (v/v) formalin (50 μL) was injected subcutaneously into the plantar surface of the left hind paw. The formalin injection-induced increase of extracellular glutamate (a), and GABA (b) from baseline. The mean concentration for the first three dialysate samples from the beginning of sample collection was defined as the baseline concentration. Values are expressed as the mean ± SEM (n = 4-5).

Supplementary Figure 3. Glutamate transporters expression in the spinal dorsal horn on day 7.
On day 7, the dorsal horn of the L4-5 spinal cord was quickly removed, and then protein expressions of GLT-1 (a), and EAAC1 (b) were analyzed by Western blot (n = 6).

Supplementary Figure 4. Unedited full bolts.

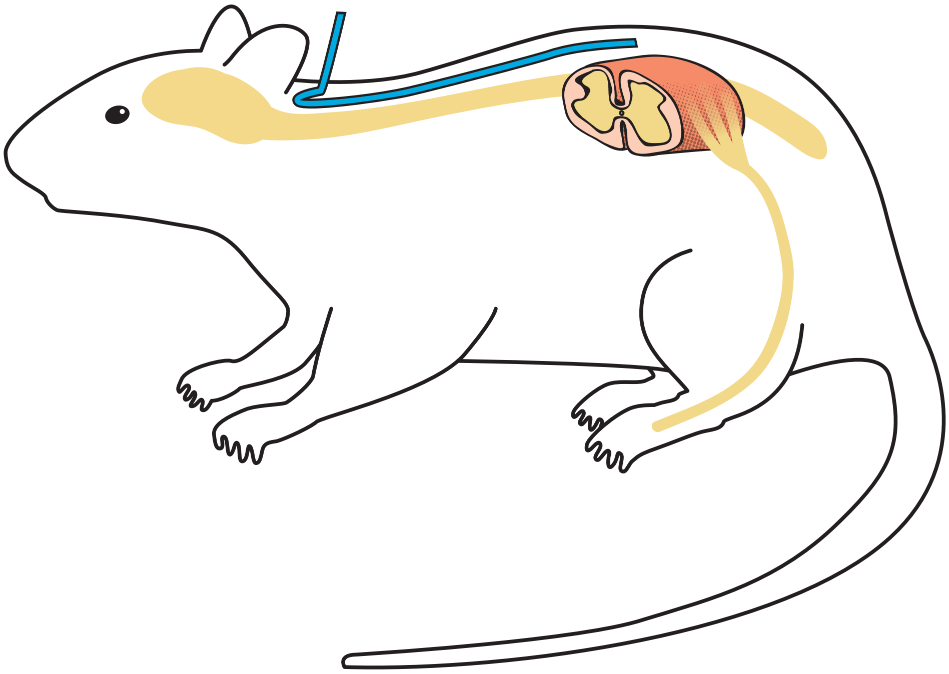

Supplementary Figure 1

a

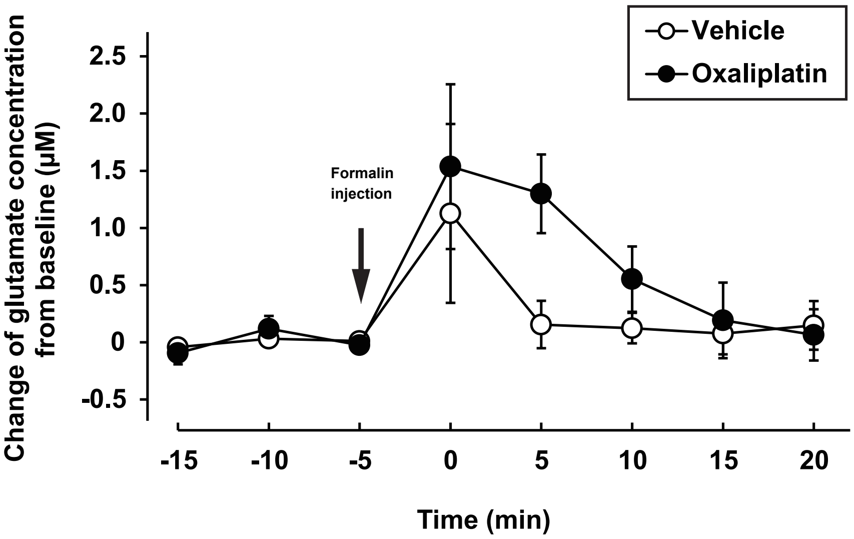

b

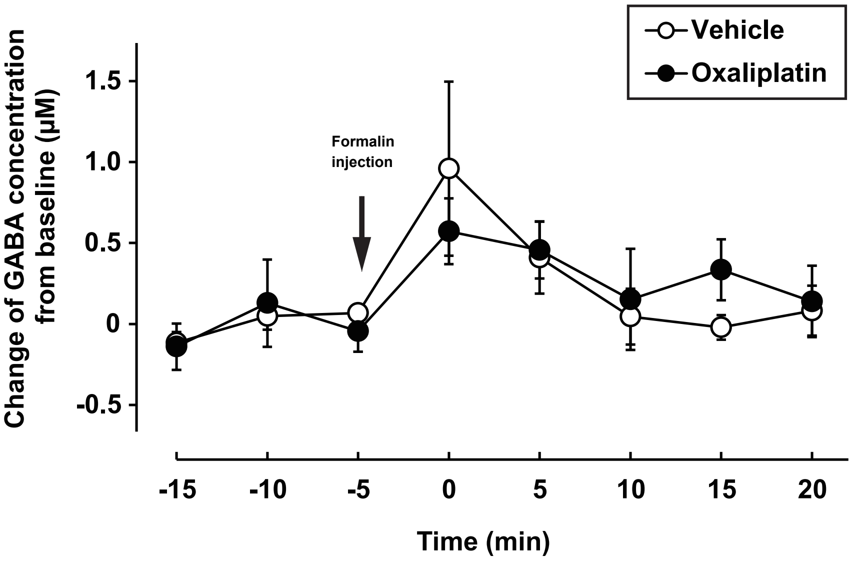

Supplementary Figure 2

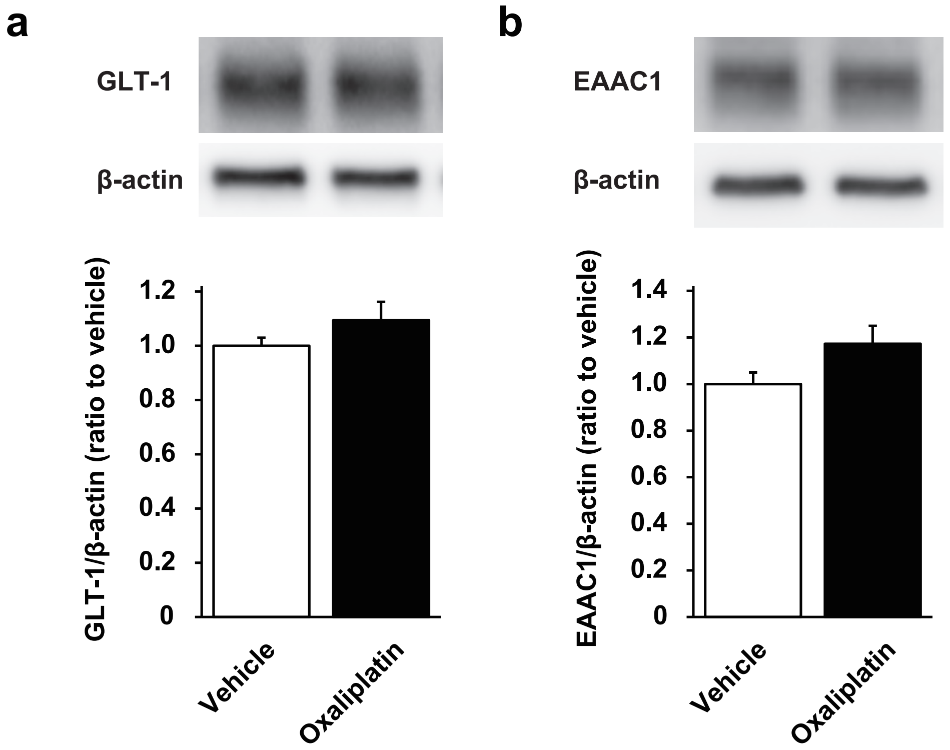

Supplementary Figure 3

Figure 2a

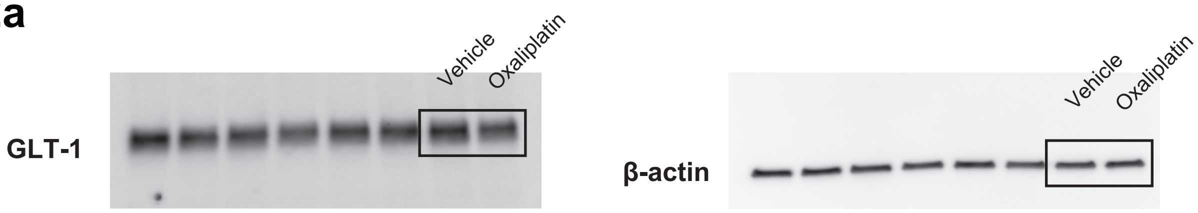

Figure 2b

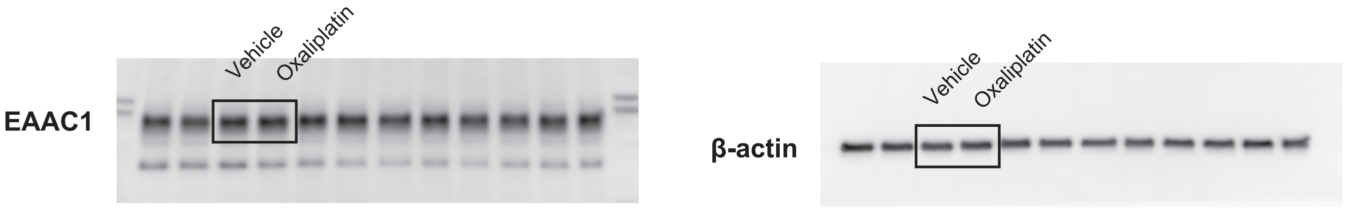

Figure 3e

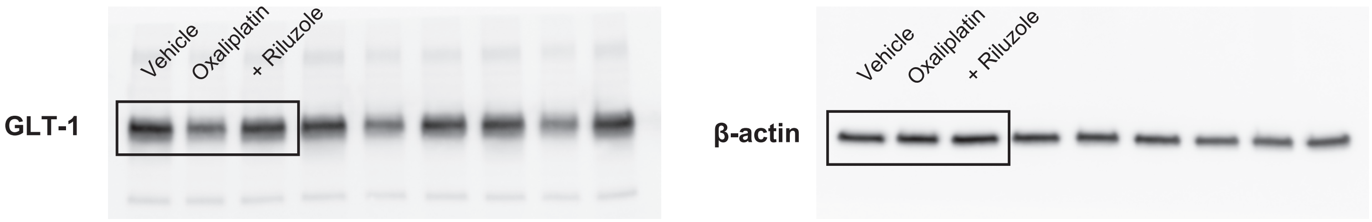

Figure 3f

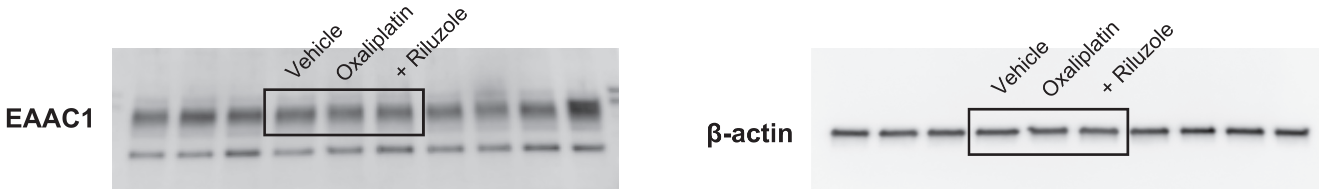

Supplementary Figure 3a

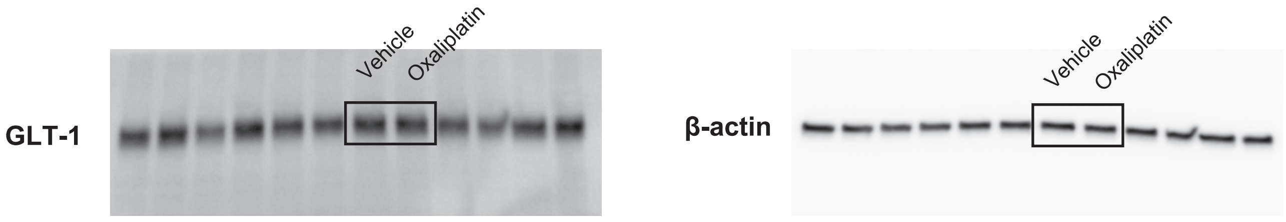

Supplementary Figure 3b

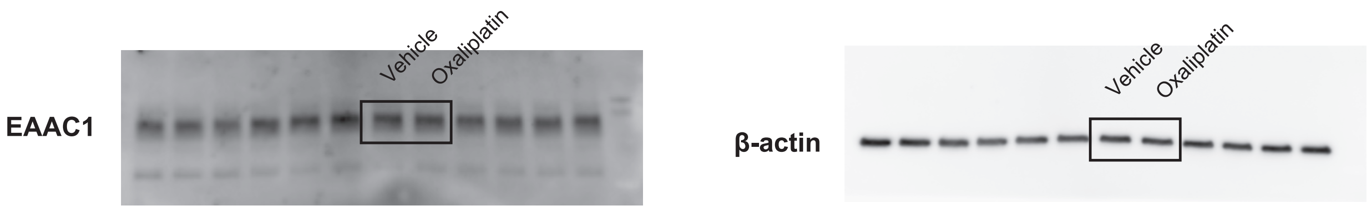

Supplementary Figure 4
